# SASFF: A Video Synthesis Algorithm for Unstructured Array Cameras Based on Symmetric Auto-Encoding and Scale Feature Fusion

**DOI:** 10.3390/s24010005

**Published:** 2023-12-19

**Authors:** Linliang Zhang, Lianshan Yan, Shuo Li, Saifei Li

**Affiliations:** 1 School of Information Science and Technology, Southwest Jiaotong University, Chengdu 611756, China; lsyan@swjtu.edu.cn (L.Y.); lishuo@my.swjtu.edu.cn (S.L.); lisaifei@swjtu.edu.cn (S.L.); 2 Shanxi Intelligent Transportation Institute Co., Ltd., Taiyuan 030036, China

**Keywords:** array cameras, video synthesis, ultra-high resolution video, image matching, deep learning

## Abstract

For the synthesis of ultra-large scene and ultra-high resolution videos, in order to obtain high-quality large-scene videos, high-quality video stitching and fusion are achieved through multi-scale unstructured array cameras. This paper proposes a network model image feature point extraction algorithm based on symmetric auto-encoding and scale feature fusion. By using the principle of symmetric auto-encoding, the hierarchical restoration of image feature location information is incorporated into the corresponding scale feature, along with deep separable convolution image feature extraction, which not only improves the performance of feature point detection but also significantly reduces the computational complexity of the network model. Based on the calculated high-precision feature point pairing information, a new image localization method is proposed based on area ratio and homography matrix scaling, which improves the speed and accuracy of the array camera image scale alignment and positioning, realizes high-definition perception of local details in large scenes, and obtains clearer synthesis effects of large scenes and high-quality stitched images. The experimental results show that the feature point extraction algorithm proposed in this paper has been experimentally compared with four typical algorithms using the HPatches dataset. The performance of feature point detection has been improved by an average of 4.9%, the performance of homography estimation has been improved by an average of 2.5%, the amount of computation has been reduced by 18%, the number of network model parameters has been reduced by 47%, and the synthesis of billion-pixel videos has been achieved, demonstrating practicality and robustness.

## 1. Introduction

In recent years, detection and recognition algorithms in the field of computer vision have made significant breakthroughs in performance [1,2,3,4,5,6,7,8,9,10]. However, the quality of images and videos is an important factor affecting the accuracy of computer vision algorithms. There is an irreconcilable contradiction between the large field of view and high-resolution details under a monocular imaging system, and it is difficult for computer vision algorithms to balance the scope of detection and recognition with the accuracy of detection and recognition [11,12]. The amount of image and video information captured by the camera directly affects the quality of images and videos. Among them, monocular cameras and multi-lens cameras are the two main ways to obtain images and videos.

The amount of information captured by a monocular camera is constrained by the number of pixels in the image sensor of the monocular camera, which makes it impossible to achieve high-quality acquisition of large scenes. Since the field of view and pixel density of the images captured by a monocular camera are inversely proportional, expanding the field of view leads to a decrease in pixel density. Therefore, a monocular camera cannot capture high-quality detailed textures in large scenes, which severely restricts the quality of the imaged videos [13]. To achieve high-resolution imaging of large scenes, a higher resolution image sensor is needed. A higher resolution image sensor necessitates a larger lens, but due to the effects of light diffraction, geometric aberration, and chromatic aberration, the imaging quality decays exponentially when the lens size of the monocular camera increases. Therefore, the image quality of the monocular camera has almost reached its limit.

Multi-lens cameras, through high-pixel-count image sensors, can combine multiple lenses of different sizes to capture high-resolution images and videos of large scenes [14]. Multi-lens cameras have the advantages of improving video resolution, expanding video scenes, increasing video frame rate, and enhancing image dynamic range, etc. In addition, multi-lens cameras can fuse images from different types of cameras to provide more accurate information display in complex scenes. For example, fusing images from visible light cameras and infrared cameras can facilitate accurate identification of target objects in scenes with different illuminations and thermal radiations. This paper conducts in-depth research on the difficult issues of synthesizing ultra-high-resolution videos of large scenes with multi-lens cameras.

Image stitching and synthesis are the core tasks of array cameras, forming a large-scene ultra-high-resolution composite image from different perspective images captured by multiple physical cameras. The positioning of images from different perspectives in the composite image, defining image scale, and implementing image rotation can easily affect the quality of image stitching. Array cameras synthesize images through physical cameras, with a maximum difference of up to six times in the focal lengths of different physical cameras, and the focal length ratio is unknown, which leads to traditional image matching methods being unable to effectively obtain the accurate positions of images from different perspectives in the composite image. For instance, the template matching algorithm based on multi-scale pyramids can calculate the image scaling ratio based on image content [15,16], achieving the calculation of focal lengths and image positioning of different cameras; however, it cannot resolve the issue of non-parallel image horizontals between different cameras, i.e., image rotation, causing the camera images in the synthesized large-scene video to be skewed and the video effect to be poor. The template matching method based on invariant moments can solve the problem of image rotation of different cameras [17,18], enabling camera image horizontal correction, but it cannot handle the issue of inconsistent and unknown focal lengths of different cameras, causing image sizes to be disordered in the synthesized large-scene video using this method, and the video effect is poor. The network model based on deep learning can solve the feature point matching problem of multi-lens cameras by simultaneously generating the feature point positions and feature descriptors of different camera images [19,20,21]. However, the spatial positional information loss during convolution and pooling operations results in poor robustness of feature point positioning and insufficient number of feature points when there is large-scale scaling, causing the synthesized large-scene video to easily have interlaced perspectives and off-setting, and the image quality is not stable enough. At the same time, deep learning methods have the common problems of deep neural networks, namely, large network parameters, complex operations, slow calculation speed, leading to slow synthesis speed of large-scene videos, high computation cost, and poor practicality [22,23].

Through research on methods such as positioning images from different perspectives in composite images, defining image scale, and image rotation, this paper addresses the issues that existing feature matching methods cannot solve, such as large gaps in image scale ratios and image rotation in array camera images. We designed a method for positioning and stitching images from unstructured array cameras, and proposed an image feature point extraction algorithm based on symmetric auto-encoding and scale feature fusion. This achieves real-time synthesis of billion-pixel videos and solves the problem that existing image registration methods cannot meet the high-precision requirements of array camera image registration. The contributions of this paper are as follows:

- We propose a novel multi-scale unstructured array camera system complemented by an efficient image positioning and stitching algorithm, enabling the synthesis of ultra-high-resolution videos for extensive scenes that surpass the limitations of single-lens cameras in resolution and detail retention.
- We propose a symmetric auto-encoder architecture that enhances feature point detection through hierarchical image feature reconstruction and layer-specific scale feature integration, and use depthwise separable convolutions to replace traditional convolutional operations.
- Our method has been rigorously tested against prevalent algorithms, showing promising results in feature point detection, homography estimation, and matching, and has demonstrated the capability to synthesize 300-million-pixel videos in real time, evidencing its practical applicability and robustness.

The rest of this paper is organized as follows: Section 2 briefly introduces related work on array cameras. Section 3 provides a detailed explanation of the design principle of multi-scale unstructured array cameras, proposes a feature point extraction algorithm based on symmetric auto-encoding and scale feature fusion, and proposes an array camera image positioning and stitching algorithm. Section 4 conducts comparative experiments on the proposed algorithms. Finally, Section 5 concludes the paper.

## 2. Related Work

To break through the bottleneck of existing single-lens camera imaging systems, researchers have proposed various array camera structural designs and image fusion algorithms based on disciplines such as bionics, optics, and computer vision. Currently, there are mainly three design schemes for wide-field ultra-high-definition array cameras: The first scheme uses the optical imaging design of multi-layer lenses to solve the diffraction and chromatic aberration problems caused by traditional single-lens cameras, and combines multiple miniature image sensors in an array to increase the area of the photosensitive elements [24,25]. This design is complex and bulky, generally used for monitoring space bodies, not suitable for highway traffic and other areas where small monitoring equipment is needed. The second scheme synthesizes panoramic images by taking sectional shots with a single camera [26], similar to the imaging method of remote sensing satellites. This panoramic stitching technology is currently very mature, but the disadvantage is that the shooting speed is slow, and there are time differences between images from different field of view areas. It can only be used to shoot static scenes; therefore, the practicality of single camera panoramic stitching technology is not strong. The third scheme uses multiple cameras combined in an array, and each camera is responsible for capturing images of different areas in a large field of view. All images captured by the cameras are transmitted in real time to a computing platform for panoramic image fusion and stitching. This scheme is an effective means to achieve the synthesis of wide-field ultra-high resolution videos [26,27,28,29,30,31,32].

Research on synthesizing ultra-high-resolution images through multi-image fusion began in the mid-20th century. Kopf et al. [26] designed an electric pan-tilt mechanism to automatically scan and capture images of different areas in a wide field of view, and then stitched them into a panoramic image. This scheme is the prototype of array camera technology, simulating the image acquisition method of a camera array by capturing images at different angles with a single camera. However, the method cannot synthesize video. In 2009, Hungarian scientist Bakos et al. [27,28] designed the HATSouth celestial monitoring system, which is the world’s first imaging system capable of monitoring celestial bodies 24 h a day throughout the year. The design comprising six identical sets of telescope equipment located at three observatories in the southern hemisphere is applicable only to the field of celestial observation. In 2010, Aoyama Gakuin University in Japan created the AROMA-W system [29], which is used for distance measurement and position calibration of planets in space. The AROMA-W system uses an array camera comprising 12 consumer-grade SLR cameras to capture the optical information of space planets. Through image fusion algorithms, ultra-high-definition images with a 45° field of view can be obtained, the field of view of the video synthesized by this method is still small and cannot be used for capturing large scenes. Wilburn et al. [30] used 100 board cameras to design a large-scale array camera system. The goal of this system is to explore a low-cost array camera system that can be practically applied, the method is complex to configure and maintain, and each camera needs to be calibrated to ensure that their viewing angles and time synchronization are accurate. Nomura et al. [31] fixed miniature cameras on flexible boards to design a portable array camera with variable angles. Although the design is portable and can be angled, the reliability and adaptability of this design are poor. Brady et al. [25] designed the AWARE-2 array camera, which is a large-scale array camera system comprising 98 miniature cameras. The 98 miniature cameras in this system are evenly distributed on a hemisphere. The system can synthesize about 1 billion pixels and 5 frames per second video. The system’s image synthesis algorithm is complex and cannot synthesize ultra-high resolution video in real time. Cossairt et al. [24] designed an array camera system with a shared front object lens, and the front object lens adopts a spherical lens optical design. The array camera optics are complex and costly. The team led by Academician Dai Qionghai of Tsinghua University took a different approach, first proposed a new theory of non-structured computational imaging, successfully developed an array image sensor with a billion-pixel intelligent imaging system [32], completed the real-time synthesis of billion-pixel videos, and achieved wide-field ultra-high-resolution video depth perception and reconstruction at the hundred-meter level. However, the method is an extension of the structured array camera, which uses a fixed focal length camera and requires customization of the corresponding array camera hardware for different scenes, which is still less practical. In summary, using array camera technology to solve the bottleneck of the field of view and resolution of a single-lens camera, and to achieve the real-time synthesis of wide-field ultra-high-resolution video, has been a hot topic of research in recent years.

## 3. Methods

### 3.1. Network Architecture for Methods

**Figure 1:**
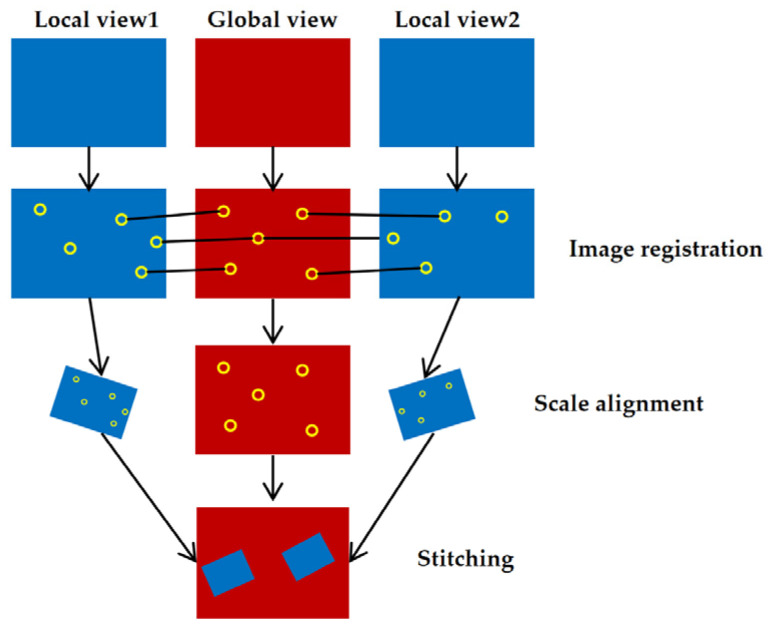
The overall architecture of the video synthesis method.

In this paper, we propose an unstructured array camera design that uses one wide-angle camera to capture large field of view global images and multiple telephoto cameras to capture local high-definition images, and utilizes an unstructured fusion algorithm that can sparsely assign local cameras to adapt to the shooting needs of different scenes. The image embedding process of the unstructured array camera is shown in Figure 1. After the global camera and multiple local cameras capture images at the same time, firstly, image feature matching and localization are carried out for each local camera image and the global camera image, respectively. Next, the scale ratio of the local camera is calculated and scaled to the same ratio for all the cameras, and then the distortion correction is carried out for the local camera image and embedded into the global image. Finally, the large field of view ultra-high resolution image is obtained.

Due to the limitations of single-lens cameras in capturing ultra-high-resolution images of large scenes, image stitching and fusion using multiple cameras require addressing challenges such as aligning images from different viewpoints, calculating image scale, and rotation. In order to achieve real-time synthesis of billion-pixel videos at a relatively low cost and overcome the effects of position, scale, and rotation in composite images while obtaining clear ultra-high-resolution images of large scenes, this paper proposes an image feature point extraction algorithm based on symmetric auto-encoding and scale feature fusion. By utilizing a shared encoder to extract deep features from the input images for matching purposes, an image feature point probability map is computed using the feature point decoder along with generating a corresponding feature point descriptor through the descriptor decoder. Based on these calculated feature points and descriptors, we propose an array camera image positioning method that employs area ratio-based scaling combined with homography matrix transformation to efficiently map each camera’s captured image onto its correct position within the composite frame. This approach enables real-time synthesis of billion-pixel videos by seamlessly integrating individual frames.

### 3.2. Imaging Method for Unstructured Array Cameras

In response to the problem that a single-lens camera sensor has limited pixels and cannot capture high-quality detailed textures in large scenes, an unstructured array camera is designed. By stitching together frames captured by multiple cameras with different viewpoints and different focal lengths within the same field of view, ultra-high-resolution and ultra-large scene video synthesis are achieved. This unstructured array camera design draws inspiration from the compound eye principle in biology.

In the field of biology, human perception information only comes from a part of the field of view. Within the 120° field of view of the human eye, only a 10° field of view is a visual-sensitive area, information can be correctly recognized within a 10° to 20° field of view, and the 20° to 30° field of view is more sensitive to moving objects. Generally, the field of view that the human eye can focus on and observe is 25°, which accounts for about 20% of the entire field of view of the human eye. Therefore, the vast majority of information in human vision comes from 20% of the area in the complete field of view.

**Figure 2:**
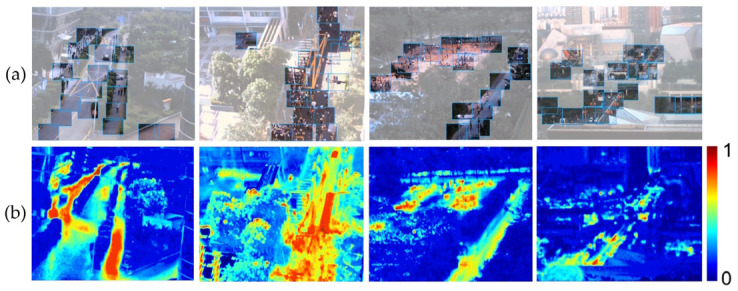
Principle of non-uniform distribution of visual signals. (**a**) Outdoor shot with a 16mm camera; (**b**) Time entropy map Corresponding to the real shot.

In the real world, visual information in broad areas also follows the principle of non-identical distribution. The field of view of a lens with a focal length of 16 mm is about 114°, and the images taken with this lens can simulate the visual range of the human eye. As shown in the real shot group (a) in Figure 2, it can be found that valuable visual signals are mainly distributed in areas where vehicles and people move, such as streets and stadiums. By calculating the content changes in the image sequence in the video, the time entropy map of group (b) in Figure 2 is obtained. The time entropy map reflects the spatial information distribution density of the scene. By observing the time entropy map, it can be found that the core information area accounts for about 25% of the entire field of view. Therefore, the unstructured array camera can make use of this principle, identify areas with visual value in the field of view through intelligent algorithms, allocate more camera field of view resources to these areas, and capture high-information videos with low-cost array cameras.

**Figure 3:**
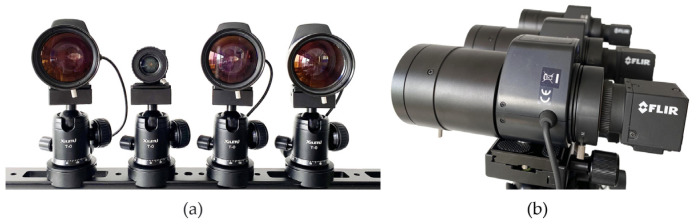
Array camera hardware platform. (**a**) Front view; (**b**) Side view.

Inspired by the above biomimetic principles, this paper proposes and designs a scalable multi-scale unstructured array camera imaging method. The unstructured array camera is designed with one wide-angle camera and three long-focal length cameras. Among them, the wide-angle camera uses a 16 mm fixed-focus lens to capture large field of view images similar to the human eye, which this paper refers to as the “global camera”. A lens with a variable focal length of 25–135 mm is used to capture high-definition images of local areas, which this paper refers to as the “local camera”. All cameras are FLIR BFSU3-120S4C-CS rolling shutter cameras, equipped with Sony IMX226 sensors, capable of capturing 4000 × 3000 resolution, 10-bit color depth images at a frequency of 30 frames per second. All cameras are mounted on a gimbal fixed on a 50 cm long rail, and each camera can freely change its viewing angle, and the three local cameras can freely adjust the lens focal length. The unstructured array camera is shown in Figure 3.

In traditional array camera systems, different cameras must capture a set of videos with overlapping fields of view. They locate matching points between different videos through image feature matching, then solve the homography matrix to get the relative positions of different cameras, and then use a planarizer for global optimization. This is a computationally intensive non-convex optimization problem. In response to the high complexity and poor practicality of image matching and stitching in traditional array cameras, this paper proposes an unstructured embedding method. Through the global camera, it can locate the field of view position of all local cameras, and no longer requires the field of view of each local camera to overlap.

The unstructured array camera uses one wide-angle camera to capture a large field of view image and multiple long-focal-length cameras to capture local high-definition images. With the use of an unstructured embedding algorithm, local cameras can be sparsely allocated to adapt to the shooting requirements of different scenes, thus achieving low-cost, high-information large field of view monitoring. Not only is the hardware cost of the unstructured array camera low, but its algorithmic process is also more efficient than that of structured array cameras.

### 3.3. Feature Point Extraction Based on Symmetric Auto-Encoding and Scale Feature Fusion

**Figure 4:**
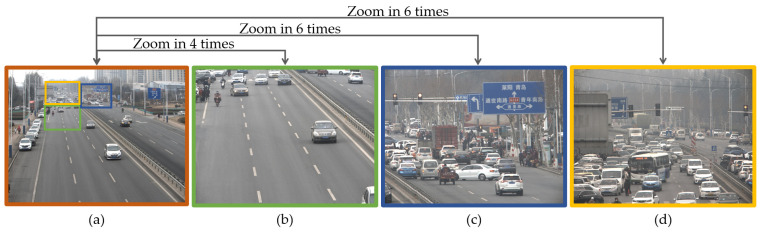
Viewpoints of each camera. (**a**) Global view; (**b**) Local view 1; (**c**) Local view 2; (**d**) Local view 3.

To synthesize the optimal large field of view ultra-high-resolution video, first adjust the global camera to the field of view area that needs to be shot, and calculate the time entropy map of the current large field of view from the video captured by the global camera. After searching for areas with high information value in the time entropy map, manually adjust the direction of the gimbal of each local camera so that the viewpoint of the local camera covers the area where the high-definition field of view needs to be captured, as shown in Figure 4.

After obtaining the images captured by each camera, it is necessary to locate and stitch the images from each camera. Feature point extraction is a core step in image location. Its goal is to extract feature points from the image that can be used for image location matching. By converting these large amounts of image data into a small number of feature points, it greatly reduces the dimensionality of the data and reduces computational complexity.

Due to the maximum focal length difference of 6 times between the global camera and the local camera, and the unknown focal length ratio, traditional image matching methods cannot effectively solve problems such as image location, scale ratio calculation, and image rotation in array cameras. For example, traditional multi-scale pyramid-based template matching algorithms can handle image matching problems at different scales [15,16], which can solve the problem of large focal length differences in array cameras. However, these methods cannot solve the problem of non-parallel image horizontals between different cameras, i.e., image rotation, which leads to skewing of the pictures of each camera in the synthesized large-scene video, resulting in poor video effects. Template matching methods based on invariant moments are invariant to image rotation and translation [17,18]; therefore, they can solve the problem of image rotation in array cameras. However, these methods cannot handle the inconsistency and unknown focal length of different cameras, resulting in disordered image sizes in the synthesized large-scene video using this method, leading to poor video effects. Currently, widely researched deep learning-based feature point extraction algorithms [19,20,21] learn and extract useful features from images automatically using convolutional neural networks, reducing the workload of feature design, and achieving better feature point extraction performance. However, deep learning-based algorithms have problems with the loss of spatial location information during convolution and pooling operations, resulting in poor robustness of feature point location capability and insufficient feature point numbers during large-scale scaling. The synthesized large-scene video is prone to staggered and offset viewing angles, and the imaging quality is not stable. For example, SuperPoint [21] is an end-to-end trained deep learning model used to detect and describe key points in images. Its main advantage is that it combines key point detection and descriptor generation into a unified network structure, allowing it to be trained from unlabeled data. However, the SuperPoint network model has the common problems of deep neural networks, namely, large network parameters, complex operations, slow computing speed, which result in slow synthesis speed of large-scene videos, high computational power consumption, and poor practicality [22,23].

**Figure 5:**
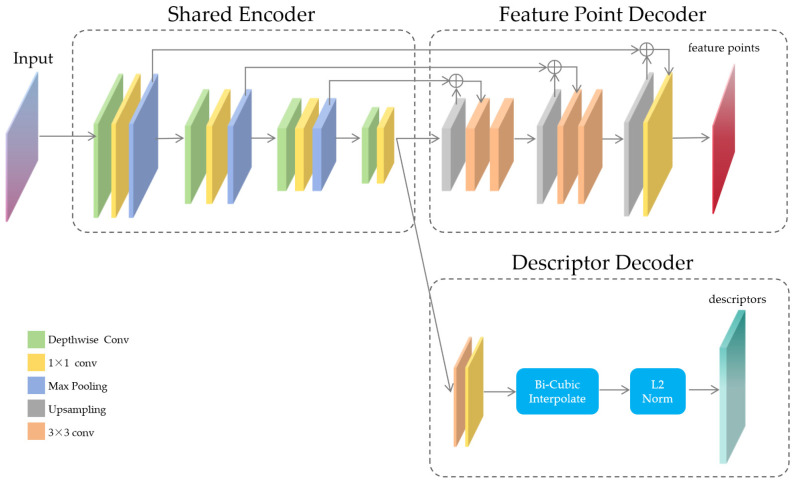
SASFF network structure.

This article optimizes the model based on the research of deep learning models and the SuperPoint network model. By adding a symmetric structure auto-encoder, it gradually restores the position information of the features during decoding and fuses multi-scale features to improve the model’s ability to locate feature points and increase the number of extracted feature points. The deep separable convolution method is optimized to enhance the model’s operation speed. The network model proposed in this article comprises a shared encoder, three max pooling layers, and two decoders. Among them, the shared encoder is used to extract image features, and then three max pooling layers are used to reduce the dimension of image features; one decoder head is used for image feature point detection, and the other is used to generate feature point descriptions. The input and output sizes of the two encoder heads are the same, which facilitates the correspondence of positions. The design of the network model is shown in Figure 5.

The data flow of this neural network model is as follows: given an input image, first scale its size to H×W, then input the processed image into the shared encoder. The shared encoder is a deep separable convolution network used to obtain the deep features of the image. The deep features of the image outputted by the shared encoder are used as inputs for both the feature point decoder and the descriptor decoder. In the feature point decoder, the position information lost in the shared encoder is restored through up-sampling operations to get the feature point probability map of the image; in the descriptor decoder, convolutional filtering and differential amplification are used to obtain the corresponding feature point descriptors of the image. The obtained feature point probability map and feature point descriptors will be used for subsequent image location and scale ratio calculations.

#### 3.3.1. Shared Encoder

The shared encoder is a convolutional neural network that takes an input image and, through a series of convolution and pooling operations, transforms the original pixel-level image data into a feature map. This feature map is a multi-channel two-dimensional array, where each channel corresponds to a specific feature of the input image. The feature point decoder uses information in the feature map to determine the key point positions in the image, while the descriptor decoder uses the feature map to generate descriptors for each key point.

**Figure 6:**
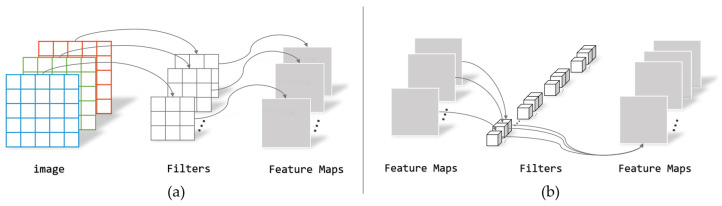
Depthwise separable convolutions. (**a**) Depthwise convolutional filters; (**b**) Pointwise convolution filters.

Existing deep learning-based feature point extraction methods mostly use VGG-like network models to extract features from input images. The VGG network model is a high-performance image feature extractor, but it has many network parameters and a large amount of computation, resulting in low efficiency in image feature point matching tasks in real-time high-resolution scenarios. To reduce the number of network model parameters and the amount of computation, and to speed up the model’s feature point extraction, this paper proposes the use of lightweight deep separable convolution [33] as a replacement for the VGG-like network model. Deep separable convolution divides traditional convolution into intra-channel and inter-channel convolutions, as shown in Figure 6. Suppose the size of the input image is H×W×C. Intra-channel convolution performs convolution operations separately on each channel using M kernels of size N×N×1, extracting feature vectors within each channel, and obtaining a total of M×C feature maps. Inter-channel convolution uses M kernels of size 1×1×1 to carry out traditional convolution operations on M×C feature maps, to extract features between different channels.

If traditional convolution operations are used, the number of parameters is C×M×N×N and the amount of computation is H×W×C×M×N×N. For deep separable convolution, when intra-channel convolution operations are used, the number of parameters is C×1×N×N, which is 1/M of the traditional convolution, and the amount of computation is H×W×C×1×N×N, which is 1/M of the traditional convolution. When inter-channel convolution operations are used, the number of parameters is C×M×1×1, which is $1/N^{2}$ of the traditional convolution, and the amount of computation is H×W×C×M×1×1, which is $1/N^{2}$ of the traditional convolution. Therefore, the number of parameters and the amount of computation for deep separable convolution is $1/M+1/N^{2}$ of traditional convolution. Compared with traditional convolution, deep separable convolution significantly reduces the amount of computation and the number of parameters in the neural network.

In response to the issue where using deep separable convolution to improve network model operation speed results in a decrease in image feature extraction capabilities, this paper proposes using 1×1 point-wise convolution operations to expand the channels of the feature map, enriching the number of features and improving the accuracy of the network model. At the same time, the feature output dimension is increased from 128 dimensions to 256 dimensions to compensate for the loss of feature information. In this paper, the size of the convolution kernel of the intra-channel convolutional layer used is 3 × 3, and the size of the convolution kernel of the inter-channel convolutional layer is 1 × 1. The convolutional steps are all 1, and the final output channel dimension of the depth-separable convolutional module is 256 × 1.

#### 3.3.2. Feature Point Decoder

After the shared encoder extracts the deep features of the image, it is necessary to identify key points in the image feature vectors. These key points are significant features of the image, such as corners, edges, etc. They provide a stable and robust relationship between images and are an important basis for array camera image stitching. In contrast to traditional image matching, image stitching requires more precise feature point locations. The pooling layers of the shared encoder lose a large amount of feature point location information while reducing the dimension of image features. To recover the location information lost in the pooling layer, the information of the three pooling layers in the shared encoder is first up-sampled using bilinear interpolation, and then the low-level feature maps at the corresponding positions are concatenated. By embedding low-level visual features as a supplement to high-level semantic features, the feature position information is gradually restored through a hierarchical restoration process. The feature point loss $L_{f}$ is a binary cross-entropy loss, the input image size is H×W×C, Y is the image feature point label, $Y^{\prime }$ is the model output value, and the calculation method of the feature point loss is as follows:

(1) $$L_{f}=\frac{1}{HW}\sum_{i=1}^{H}\sum_{j=0}^{W}Y_{ij}\log{\hat{Y}}_{ij}+(1-Y_{ij})\log(1-{\hat{Y}}_{ij}),$$

The output of the feature point decoder is a list of keypoint locations, each corresponding to the pixel at the same position in the input image. At the same time, each detected keypoint is assigned a probability value, representing the probability that the location is a keypoint. The output of the feature point decoder can be viewed as a probability map, where each pixel’s value represents the probability that the pixel in the original image is a keypoint.

#### 3.3.3. Descriptor Decoder

The descriptor decoder generates an independent descriptor for each detected feature point. A descriptor is a vector that represents the pixel pattern around a feature point, and it serves as the basis for feature matching. In tasks such as image registration, object recognition, and visual localization, the descriptors in two images are compared to find corresponding feature points, thereby determining the spatial relationship between images. Therefore, the performance of the descriptor decoder is crucial for the entire feature point extraction task. The descriptor decoder also uses the feature map generated by the shared encoder as input, then generates a descriptor at each keypoint location, ensuring that the generated descriptors are robust to small displacements, rotations, and scale changes in the image. The descriptor decoder first uses a convolutional kernel of size 3×3×128 to filter the features output by the shared encoder, and for an image of size H×W, a feature vector of size $H_{p}\times W_{p}\times 256$ is obtained. Then, a convolutional kernel of size 1×1 is used to obtain a feature vector of size $H_{p}\times W_{p}\times L_{D}$. To have a size matching the output image, the size is restored using bicubic interpolation, and the $L_{2}$ normalization activation is used to obtain a $H\times W\times L_{D}$ feature descriptor, where $L_{D}=256$ is the descriptor length. The descriptor decoder loss is made up of all cell pairs from the input image, with each cell from the original image denoted as (x,y), each cell from the transformed image denoted as $\left(x^{\prime },y^{\prime }\right)$, the similarity between the cells is $s_{xyx^{\prime }y^{\prime }}$, and $c_{xy}$, $c_{x^{\prime }y^{\prime }}$ are the center coordinates of (x,y) and $\left(x^{\prime },y^{\prime }\right)$, respectively, as follows:

(2) $$s_{xyx^{\prime }y^{\prime }}=\left\{\begin{array}{c} 1,if\left\|hc_{xy}-c_{x^{\prime }y^{\prime }}\right\|\\ 0,otherwise \end{array}\right.,$$

The calculation method for the descriptor decoder’s loss is as follows:

(3) $$L_{d}(D,D^{\prime },S)=\frac{1}{{\left(H_{p}W_{p}\right)}^{2}}\sum_{\begin{array}{l} x=1\\ y=1 \end{array}}^{H_{p},W_{p}}\sum_{\begin{array}{l} x^{\prime }=1\\ y^{\prime }=1 \end{array}}^{H_{p}^{\prime },W_{p}^{\prime }}l_{d}(d_{xy},d_{xy}^{\prime };s_{xyx^{\prime }y^{\prime }}),$$

The output of the descriptor decoder is a vector of dimension n. This n-dimensional vector is the descriptor of the feature point, which contains information about the image region around the feature point, and is used for subsequent image feature point matching.

#### 3.3.4. Loss Function

In this network model, a loss function is used to measure the difference between the model’s predicted results and the true values. By minimizing the loss function, the model can learn the mapping relationship from the input image to the target output (feature points and their corresponding descriptors). The loss function of the network model comprises the feature point decoder loss and the descriptor decoder loss. The feature point decoder loss function is used to calculate the Euclidean distance between the predicted feature point locations and the actual feature point locations. By minimizing this part of the loss, the model can learn how to accurately detect feature points in the image. The descriptor decoder loss is used to calculate the Euclidean distance between the predicted descriptors and the actual descriptors. By minimizing this part of the loss, the model can learn how to generate descriptors with strong uniqueness and robustness.

Assuming the training image X is transformed to generate image $X^{\prime }=hX$, where h is a randomly generated homography transformation matrix, $h\in R^{3\times 3}$. The original image X and the transformed image $X^{\prime }$ are inputted into the network simultaneously, D is the descriptor label, Y is the feature point label, $D^{\prime }$ and $Y^{\prime }$ are the corresponding model output values, and λ is the parameter to balance the two losses. The final loss function is:

(4) $$L(X,X^{\prime },D,D^{\prime };Y,Y^{\prime },h)=L_{f}(X,Y)+L_{f}(X^{\prime },Y^{\prime })+\lambda L_{d}(D,D^{\prime },h),$$

#### 3.3.5. Training Parameter Optimization and Transfer Learning

The SuperPoint network model uses constrained random homography transformations in self-supervised training to construct image pairs with known pose relationships, and then uses these image pairs to learn feature point extraction and descriptor generation. The basic process is to first detect a set of feature points from the target through the basic feature point detector, then apply the random homography matrix to the mapping of the input image, and finally combine all the feature points of the mapped images. I represents the input image, x is the calculated feature points, and H is a random homography matrix. The random homography transformation can be represented as:

(5) $$x=H^{-1}f_{\theta }(H(I)),$$

The SuperPoint network model is a model designed for image feature point extraction tasks in SLAM (Simultaneous Localization and Mapping). In SLAM tasks, the changes in images are characterized by large rotational variations and small scale variations. Therefore, the scale transformation in the random homography transformation is restricted to 0.8–1.2, and the rotation transformation is restricted from −180° to 180°. In unstructured array cameras, the horizon angle difference between the global camera and the local camera is generally within 10°. This paper changes the rotation restriction of the real image random homography transformation in the SuperPoint network model from −15° to 15°. The focal length ratio of the global camera and the local camera is generally 3 to 6 times, but because the image resolution in the training dataset MS COCO is around 600×600, an overly large-scale range would cause the image resolution to be quite low to extract image feature points; therefore, this paper changes the image scale restriction in the SuperPoint network model to 0.3–1.7 to adapt to the characteristics of images in the array camera. At the same time, due to the increase in the scale ratio, this paper also expands the area restriction for random image movement. Detailed improvements on the training parameters are shown in Table 1.

**Figure 7:**
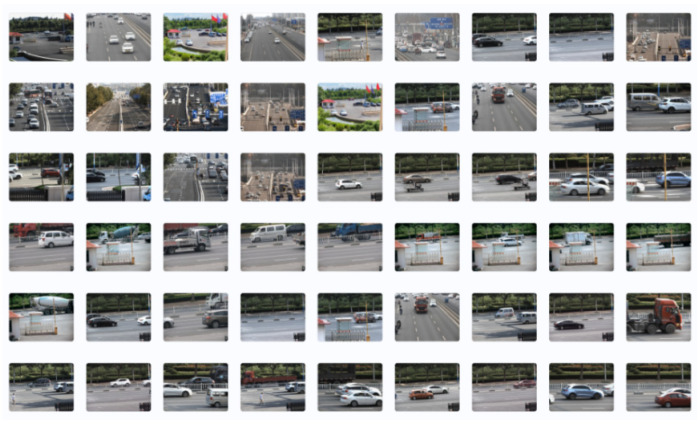
Wide field of view images in outdoor environments.

In the MS COCO dataset, there are mostly small scenes with single type objects, and the image size is relatively low. To ensure that the model trained has good feature point extraction performance in large outdoor scenes, this paper collected 5000 images with a resolution of 4000 × 3000 in three different outdoor scenes using five different focal lengths, and carried out transfer training on the well-trained network model. The collected images are shown in Figure 7.

This section provides a detailed description of the proposed feature point extraction method based on symmetric self-encoding and scale feature fusion. By using depthwise separable convolution to replace the VGG network model, the number of model parameters and the amount of computation are significantly reduced, making the model faster in inference time. At the same time, due to the reduction in the number of parameters, the model using depthwise separable convolution is less prone to overfitting than the VGG network, demonstrating better generalization ability. This section designs a feature point extraction method based on symmetric self-encoding and scale feature fusion. The symmetric structure enhances the interpretability of the model, as the representation of the hidden layer can be clearly mapped back to the input space through the decoder, allowing the model to better learn and understand the feature point information of the image; scale feature fusion embeds the deep image features extracted by depthwise separable convolution into low-level visual features, gradually restores feature point position information through hierarchical restoration, and can obtain feature representations with rich semantic information and high spatial resolution. At the same time, this section optimizes network model training parameters for the characteristics of array camera images, and uses high-resolution images taken with an array camera for transfer learning to improve the model’s feature point detection performance.

### 3.4. Optimization of Image Localization and Scale Alignment

After obtaining the set of feature point matches between the local camera image and the global camera image, it is necessary to solve for the corresponding positional relationship. According to the principles of lens imaging, if the projection mapping matrix from a point P(X,Y,Z) in real space to a point p(x,y) on the image sensor is denoted as p(x,y), then point P, point p, and the projection mapping matrix R have the following relationship:

(6) $$\left[\begin{array}{c} x\\ y\\ 1 \end{array}\right]=\left[\begin{array}{cccc} r_{11} & r_{12} & r_{13} & r_{14}\\ r_{21} & r_{22} & r_{23} & r_{24}\\ r_{31} & r_{32} & r_{33} & r_{34} \end{array}\right]\left[\begin{array}{c} X\\ Y\\ Z\\ 1 \end{array}\right],$$

In real space, there is a surjective relationship between point P(X,Y,Z) and point p(x,y) projected onto the image sensor, namely, starting from point p and drawing a ray in the direction of point P, all points in real space on this ray will be projected onto point p on the image sensor. Therefore, the projection mapping matrix R can be simplified by selecting a specific plane in real space. Here, we choose plane Z=0 in the real-world coordinate system, and the mapping relationship can be simplified as follows:

(7) $$\left[\begin{array}{c} x\\ y\\ 1 \end{array}\right]=\left[\begin{array}{cccc} r_{11} & r_{12} & r_{13} & r_{14}\\ r_{21} & r_{22} & r_{23} & r_{24}\\ r_{31} & r_{32} & r_{33} & r_{34} \end{array}\right]\left[\begin{array}{c} X\\ Y\\ Z\\ 1 \end{array}\right]=\left[\begin{array}{ccc} r_{11} & r_{12} & r_{13}\\ r_{21} & r_{22} & r_{23}\\ r_{31} & r_{32} & r_{33} \end{array}\right]\left[\begin{array}{c} X\\ Y\\ 1 \end{array}\right],$$

At this point, the real-world space is simplified to a plane parallel to the image sensor, and the points on this plane have a one-to-one mapping relationship with the points projected onto the image sensor. The projection matrix is represented by H, namely:

(8) x=HX,

(9) $$H=\left[\begin{array}{ccc} r_{11} & r_{12} & r_{13}\\ r_{21} & r_{22} & r_{23}\\ r_{31} & r_{32} & r_{33} \end{array}\right],$$

By reducing the real-world space to a plane, the projection transformation relationship between this plane and the image sensor plane is called a homography transformation, and matrix H is called a homography matrix. Further extending the homography transformation, the projection transformation relationship between two image sensor planes can be obtained. When two cameras capture the same real-world plane image at the same time, we have:

(10) $$m_{1}=H_{1}M,$$

(11) $$m_{2}=H_{2}M,$$

We can obtain:

(12) $$m_{2}=H_{2}M=H_{2}(H_{1}^{-1}m_{1})=H_{21}m_{1},$$

The homography matrix $H_{21}$ represents the image projection mapping matrix from the plane where camera A is located to the plane where camera B is located.

By setting the last element of matrix $H_{21}$ to 1, we have:

(13) $$\left[\begin{array}{c} x_{2}\\ y_{2}\\ 1 \end{array}\right]=\left[\begin{array}{ccc} h_{11} & h_{12} & h_{13}\\ h_{21} & h_{22} & h_{23}\\ h_{31} & h_{32} & 1 \end{array}\right]\left[\begin{array}{c} x_{1}\\ y_{1}\\ 1 \end{array}\right],$$

After expanding the matrix, we obtain:

(14) $$x_{2}=\frac{x_{1}h_{11}+y_{1}h_{12}+h_{13}}{x_{1}h_{31}+y_{1}h_{32}+1},$$

(15) $$y_{2}=\frac{x_{1}h_{21}+y_{1}h_{22}+h_{23}}{x_{1}h_{31}+y_{1}h_{32}+1},$$

**Figure 8:**
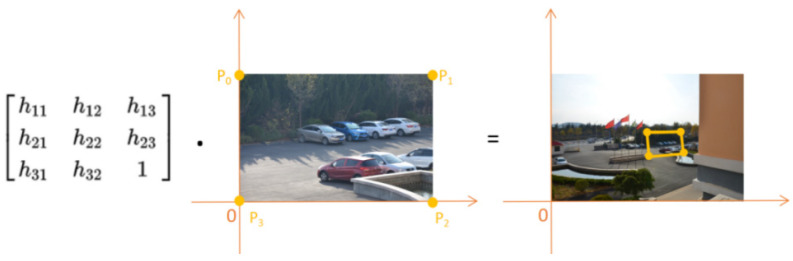
Localization of the local camera’s field of view.

After calculating the homography matrix that transforms the local camera image to the global camera image, this paper uses the lower left vertex of the local camera image as the origin of the coordinate system, and constructs a Cartesian coordinate system with the local camera image as the first quadrant of the coordinate system. By left-multiplying the coordinates of the four vertices of the local camera image by the homography matrix, the coordinates of the local camera image mapped to the global camera image can be obtained, as shown in Figure 8.

Since the image after homography transformation must be a convex quadrilateral, the scale relationship between the two can be calculated using the ratio of the area of the transformed convex quadrilateral to the area of the global image. This section proposes an area calculation method based on the coordinates of the four vertices of the convex quadrilateral, with the formula as follows:

(16) $$Area\_L=\frac{1}{2}\sum_{i=1}^{n}(P[(i+1)\%n].x\cdot P[i].y-P[(i+1)\%n].y\cdot P[i].x),$$

Here, Area_L represents the area of the transformed local camera image, P[i].x and P[i].y represent the horizontal and vertical coordinates of the four vertices of the transformed local camera image, and n=4. The final scale ratio between the global camera image and a particular local camera image is obtained as:

(17) $$scale\_ratio[i]=\frac{Area\_G}{Area\_L[i]},$$

Here,Area_G represents the area of the global camera image. This method allows for the quick and accurate calculation of the scale ratio between different cameras.

Since each local camera has a different focal length, the images captured are also of different scales. In order not to lose image information when embedding, and to avoid redundant information, all images captured by the cameras need to be adjusted to the same scale before they can be embedded and fused. Since there can be a maximum scale difference of up to 6 times between the global camera and the local camera, to reduce jagged edges that can occur after enlarging the global camera image, this paper uses a bilinear interpolation algorithm to enlarge the image.

**Figure 9:**
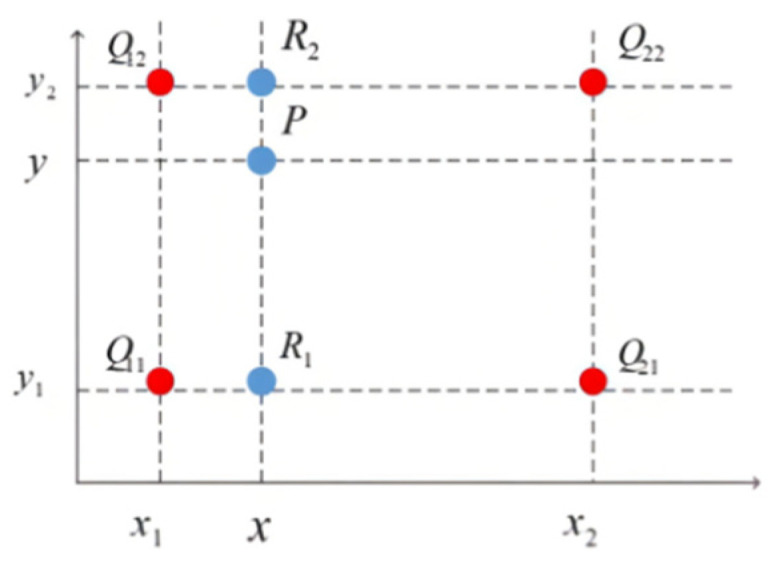
Bilinear interpolation.

The main idea of the bilinear interpolation algorithm is to calculate the pixel points added after enlarging by using the pixel values of the adjacent 4 points in the original image, as shown in Figure 9.

For the pixel points to be added, the pixel values of the four surrounding pixel points $Q_{11}(x_{1},y_{1})$, $Q_{12}(x_{1},y_{2})$, $Q_{21}(x_{2},y_{1})$, and $Q_{22}(x_{2},y_{2})$ are $f(Q_{11})$, $f(Q_{12})$, $f(Q_{21})$, and $f(Q_{22})$, respectively. The bilinear interpolation algorithm first performs interpolation twice in the horizontal direction:

(18) $$f(R_{1})=\frac{x_{2}-x}{x_{2}-x_{1}}f(Q_{11})+\frac{x_{1}-x}{x_{1}-x_{2}}f(Q_{21}),$$

(19) $$f(R_{2})=\frac{x_{2}-x}{x_{2}-x_{1}}f(Q_{12})+\frac{x_{1}-x}{x_{1}-x_{2}}f(Q_{22}),$$

Then, an interpolation operation in the vertical direction is performed:

(20) $$f(P)=\frac{y_{2}-y}{y_{2}-y_{1}}f(R_{1})+\frac{y_{1}-y}{y_{1}-y_{2}}f(R_{2}),$$

Next, the maximum value among all the scale ratios of the local camera and the global camera is denoted as max_ratio:

(21) max_ratio=Max(scale_ratio),

Then, the global camera image is enlarged by max_ratio times using the bilinear interpolation algorithm. The enlargement ratio corresponding to different local camera images is denoted as resize_ratio, and calculated as follows:

(22) $$resize\_ratio[i]=\frac{max\_ratio}{scale\_ratio[i]},$$

All local camera images are enlarged using the bilinear interpolation method. At this point, the global camera image and all local camera images are at the same scale.

Since different camera images have different scale ratios, all image scales need to be aligned before image stitching can be performed. However, after the image is scaled, the original homography matrix H is no longer valid. To reposition the scaled image, this section proposes a homography matrix scaling algorithm with scale changes.

Let the homography transformation matrix from each local camera image to the global camera image be H[i], the global camera image be $I_{global}$, the mapping matrix for enlarging the global camera image be $S_{global}$, the enlarged image of the global camera be $I_{global}^{\prime }$, the local camera image be $I_{local}[i]$, the mapping matrix for enlarging the local camera image be $S_{local}[i]$, and the enlarged image of the local camera be $I_{local}^{\prime }[i]$. For the global camera image, we have:

(23) $$I_{global}^{\prime }=S_{global}\cdot I_{global},$$

(24) $$S_{global}=\left[\begin{array}{ccc} max\_ratio & 0 & 0\\ 0 & max\_ratio & 0\\ 0 & 0 & 1 \end{array}\right],$$

Similarly, for the local camera image, we have:

(25) $$I_{local}^{\prime }[i]=S_{local}[i]\cdot I_{local}[i],$$

(26) $$S_{local}[i]=\left[\begin{array}{ccc} resize\_ratio[i] & 0 & 0\\ 0 & resize\_ratio[i] & 0\\ 0 & 0 & 1 \end{array}\right],$$

From $I_{local}^{\prime }[i]=S_{local}[i]\cdot I_{local}[i]$, we get:

(27) $$\begin{array}{cc} I_{global}^{\prime } & =S_{global}\cdot I_{global}\\ & =S_{global}\cdot H[i]\cdot I_{local}[i]\\ & =S_{global}\cdot H[i]\cdot S_{local}^{-1}\cdot S_{local}[i]\cdot I_{local}[i]\\ & =(S_{global}\cdot H[i]\cdot S_{local}^{-1}[i])\cdot I_{local}^{\prime }[i] \end{array},$$

Let S[i] be the mapping matrix that transforms the local camera image to the global camera image at the same scale, we have:

(28) $$I_{global}^{\prime }=S_{global}\cdot I_{global}=S[i]\cdot I_{local}^{\prime }[i],$$

We can get:

(29) $$\begin{array}{cc} S[i] & =S_{global}\cdot H[i]\cdot S_{local}^{-1}[i]\\ & =\left[\begin{array}{ccc} max\_ratio & 0 & 0\\ 0 & max\_ratio & 0\\ 0 & 0 & 1 \end{array}\right]\cdot H[i]\cdot {\left[\begin{array}{ccc} resize\_ratio[i] & 0 & 0\\ 0 & resize\_ratio[i] & 0\\ 0 & 0 & 1 \end{array}\right]}^{-1} \end{array},$$

For the problem of calculating the scale ratio of array camera images, this section proposes a method based on the area ratio of a convex quadrilateral, which can quickly calculate the scale ratio relationship between different cameras based on the homography matrix. For the positioning problem of array camera images, this section proposes a homography matrix scaling algorithm with scale changes. By decomposing and performing matrix operations on the homography matrix H, a calculation method is obtained that quickly positions the scaled camera image in the final ultra-large scene image. With this formula, the image position of different cameras can be quickly located after scaling, without the need for additional feature point extraction and positioning on high-resolution images, thus improving the speed and accuracy of image positioning.

### 3.5. Image Stitching Optimization

**Figure 10:**
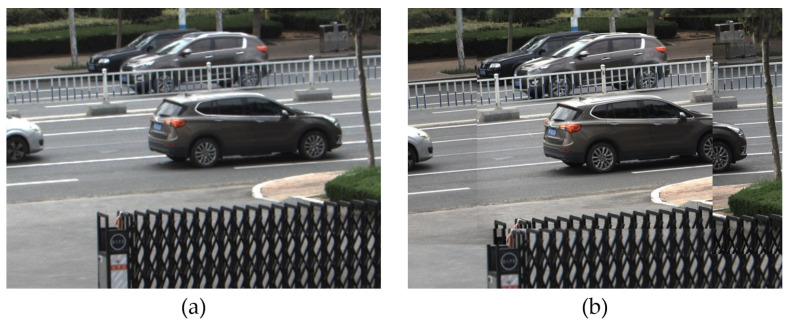
Segmentation phenomenon caused by directly embedding local camera images. (**a**) Global camera image; (**b**) Embed local camera image directly.

After obtaining the positions of the images from each camera, all the images captured by the cameras can be stitched together to form a super high-resolution image. However, due to the different physical spatial positions of each camera, they have different shooting angles when shooting the same physical plane, resulting in images with different degrees of distortion and deformation. If the local camera images are directly embedded into the global camera image, the stitched edge parts will have a clear segmentation, as shown in Figure 10. Since the field of view of all local cameras is within the field of view of the global camera, this section proposes a stitching optimization algorithm based on second image positioning. Taking the viewpoint of the global camera as the benchmark, it transforms the viewpoint of all local cameras into the viewpoint of the global camera, solving the image distortion problem caused by different camera viewpoints.

To build an image stitching model, the first step is to use perspective transformation to describe the change in viewpoint in three-dimensional space, the expression is:

(30) $$x^{\prime }=Tx,$$

(31) $$\left[\begin{array}{ccc} x^{\prime } & y^{\prime } & w \end{array}\right]=\left[\begin{array}{ccc} x & y & w \end{array}\right]\left[\begin{array}{ccc} a_{11} & a_{12} & a_{13}\\ a_{21} & a_{22} & a_{23}\\ a_{31} & a_{32} & a_{33} \end{array}\right],$$

Analysis and Decomposition of the Perspective Transformation Matrix:

(32) $$T=\left[\begin{array}{ccc} a_{11} & a_{12} & a_{13}\\ a_{21} & a_{22} & a_{23}\\ a_{31} & a_{32} & a_{33} \end{array}\right]=\left[\begin{array}{cc} T_{1} & T_{2}\\ T_{3} & a_{33} \end{array}\right],$$

In this context, $T_{1}$ represents the linear transformation of the image, $T_{2}$ represents the perspective transformation of the image, and $T_{3}$ represents the translational transformation of the image:

(33) $$\begin{array}{l} T_{1}=\left[\begin{array}{cc} a_{11} & a_{12}\\ a_{21} & a_{22} \end{array}\right]\\ T_{2}={\left[\begin{array}{cc} a_{13} & a_{23} \end{array}\right]}^{T},\\ T_{3}=\left[\begin{array}{cc} a_{31} & a_{32} \end{array}\right] \end{array},$$

Formulas (9) and (10) can be used to calculate the perspective plane transformation matrix T for the local camera and global camera, i.e., the transformation matrix T is calculated using the feature point matching set of the local camera image and the global camera image. Since the pixel density of the original image has changed after the global camera image has been interpolated and enlarged, re-extraction and matching of feature points can help achieve better results. Section 3.2 obtained the position of the local camera in the global camera; therefore, it is only necessary to extract and match feature points between the local camera image and the sub-image of the global image at the corresponding position, and then calculate the perspective transformation matrix T of the local camera image. This will yield a more precise image viewpoint correction stitching matrix.

This section proposes a stitching optimization algorithm based on secondary image positioning to address the image distortion problem caused by the difference in viewpoints of array cameras. By extracting and matching feature points between the local camera image and the sub-image of the global image at the corresponding position, not only is the computation amount of feature point extraction reduced, but also errors in matching in featureless areas of the global camera image are avoided. This enhances the accuracy of feature point matching and effectively improves the effect of image stitching.

## 4. Results and Discussion

This section compares the performance of the feature point extraction algorithm proposed in this paper and several commonly used feature point detection algorithms through objective quantification experiments and subjective assessment experiments. The hardware platform used is a PC with an Intel i5-8400 processor, 16 GB of memory, and a 500 GB solid-state drive. The software framework used is Python 3.8.

### 4.1. Experimental Settings

#### 4.1.1. Benchmark Experiment

In this section, several typical feature point extraction algorithms are used to conduct benchmark performance testing on the HPatches dataset [34]. The HPatches dataset is a real-world image dataset used for testing the performance of feature point extraction and descriptor generation. The HPatches dataset is divided into two parts: illumination change detection, which includes 343 pictures under 57 scenes and viewpoint change detection, which includes 354 pictures under 59 scenes.

#### 4.1.2. Benchmark Feature Point Detection Performance Comparison on HPatches Dataset

This section compares three classic feature point extraction algorithms, FAST [35], Harris [36], and Shi [37], with the SuperPoint network model based on deep learning. The FAST (Features from Accelerated Segment Test) algorithm is a method of feature point detection used for real-time image processing. This algorithm first selects a pixel and then checks the 16 pixels around it. If there are a continuous n pixels in these 16 pixels that are either significantly darker or brighter than the center pixel, then the center pixel is considered a feature point. This pattern detection method allows the FAST algorithm to quickly and effectively find feature points in an image. The Harris algorithm first calculates the gradient of the grayscale intensity of the image, and then builds a 2 × 2 matrix formed by the outer product of the gradient. The eigenvalues of this matrix can be used to assess whether each pixel in the image is a feature point. If the two eigenvalues are both large, then this pixel is considered a feature point. The Shi feature point detection algorithm is a commonly used feature detection method in computer vision. It is an improved version of the Harris corner detector, with the main difference being that it uses a simpler and more intuitive corner response function. SuperPoint is a machine learning-driven feature point detection and descriptor extraction algorithm. During the training process, the network is first pre-trained on synthetic data and then fine tuned on real data. This allows SuperPoint to learn effective feature point detection and descriptor generation strategies without manually annotated training data.

#### 4.1.3. Comparison of Homography Estimation Accuracy

This section compares the homography estimation benchmark experiments of three classic feature point extraction algorithms, ORB [38], SIFT [39], and SURF [40], and the SuperPoint network model based on deep learning. The ORB (Oriented FAST and Rotated BRIEF) algorithm is an efficient method for feature detection and descriptor generation, combining the speed advantage of the FAST corner detector and the rotation invariance of the BRIEF descriptor; SIFT (Scale-Invariant Feature Transform) is a widely used feature detection and description algorithm in computer vision, capable of maintaining stability under conditions such as image scaling, rotation, and brightness changes; SURF (Speeded Up Robust Features) is a fast and robust feature detection and description algorithm, aiming to provide more efficient performance than SIFT while maintaining similar characteristics, such as robustness to scale, rotation, and brightness changes.

#### 4.1.4. Viewpoint Correction Experiment

In this section, we used real scene images from an array camera to compare the feature point extraction effects of different feature point extraction algorithms on images with about five times scale difference. The comparison methods include three classical feature point extraction algorithms, ORB, SIFT, and SURF, and three advanced feature point extraction algorithms, LoFTR [41], DISK [42], and LightGlue [23]. The performance of different algorithms is evaluated by intuitively observing the image feature point matching effects.

#### 4.1.5. Video Synthesis Experiment

To verify the effectiveness of the algorithm proposed in this paper, we conducted an on-site video synthesis test under an outdoor road scenario. The system is able to synthesize in real time, 17 frames per second of ultra-high-resolution video with about 300 million pixels in a wide field of view.

### 4.2. Results and Discussion

#### 4.2.1. Comparison of Parameter Quantity and Operation Quantity

The statistical data of the parameter quantity and operation quantity of the original SuperPoint network model and the network model proposed in this paper are shown in Table 2. The experimental results show that the parameter quantity of the network model proposed in this paper is 47% less than that of the SuperPoint network model, and the operation quantity is 18% less than the original SuperPoint network model. Therefore, depthwise separable convolution can greatly reduce the parameter quantity and operation quantity of the deep neural network model, and enhance the speed of feature point extraction. At the same time, in depthwise separable convolution, channel-wise convolution is performed first, followed by cross-channel convolution, which enables the model to better utilize the information of the feature map and has better generalization ability. The lower number of parameters and operations can speed up image feature point extraction and matching, and improve the frame rate of ultra-high resolution video synthesis.

#### 4.2.2. Benchmark Feature Point Detection Performance Comparison on HPatches Dataset

The results of the comparison experiment are shown in Table 3. As can be seen from the experimental results in Table 3, the network model proposed in this paper achieves the best results in both benchmark tasks. Compared with traditional feature point extraction algorithms, the method proposed in this paper can learn a wealth of change patterns from a large amount of training data, including scale changes, rotation, illumination changes, etc. This enables it to still detect stable feature points and generate robust descriptors under these changes. Compared with SuperPoint, the method proposed in this paper improves the average performance of the HPatches benchmark feature point detection experiment by 5%. This is due to the fact that the symmetric structure enhances the interpretability of the model, and the representations in the hidden layers can be clearly mapped back to the input space through the decoder, enabling the model to better learn and understand the feature point information of the image. At the same time, the scale feature fusion gradually restores the feature point position information through hierarchical restoration, thus obtaining a feature representation with rich semantic information and higher spatial resolution, effectively improving the model’s recognition ability. The excellent feature point detection performance helps the model to obtain accurate feature point matching in an array camera with a five-fold difference in scale ratio, which can be computed to obtain more accurate local image localization and scale ratio.

#### 4.2.3. Comparison of Homography Estimation Accuracy

The experimental results are shown in Table 4. The experiments show that the feature point detection algorithm proposed in this paper maintains good homography estimation accuracy while significantly reducing the parameter quantity and computational quantity of the deep neural network model. The homography estimation can reflect the aberration caused by the difference of view angles when matching images, a good accuracy of homography estimation can help to achieve a better aberration correction effect, and the spliced image is less prone to crack phenomenon.

#### 4.2.4. Viewpoint Correction Experiment

**Figure 11:**
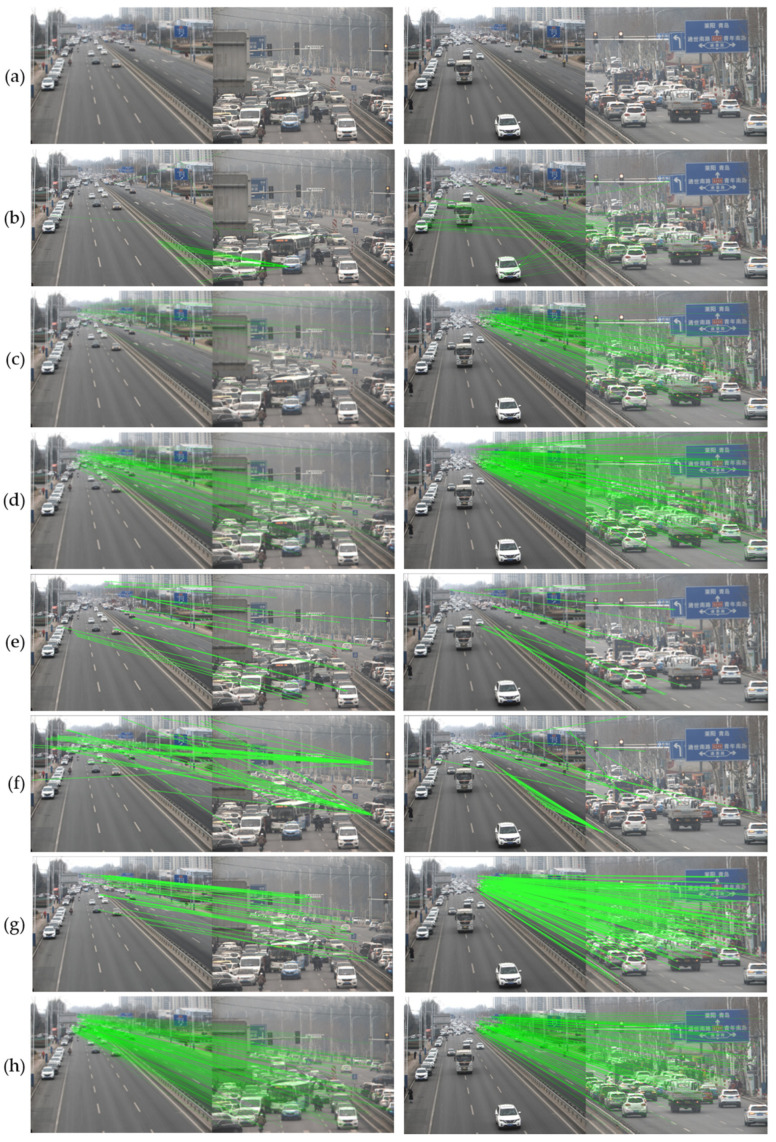
Comparison of feature point matching performance. (**a**) Origin; (**b**) ORB; (**c**) SURF; (**d**) SIFT; (**e**) LoFTR; (**f**) DISK; (**g**) LightGlue; (**h**) Our.

The comparison results are shown in Figure 11. As can be seen from the original images in group (a), the area of the local camera image with a five times scale difference accounts for about 4% of the global camera image, which has a significant scale difference visually. In group (b) images, the ORB algorithm has problems with incorrect feature point pair matching in both test image groups, specifically, the first group of images matched to the wrong area, and the matching results of the second group of images are completely scattered pairs of points. In the images of group (c), the SURF algorithm matched the correct pairs of feature points, but the number is quite small to effectively calculate the viewpoint correction matrix. In the images of group (d), the SIFT algorithm matched more pairs of feature points, but a large number of incorrect matches occurred in both groups of test images, and most of the incorrectly matched feature points appeared in the edge area of the local camera image. In group (e) and group (f), LoFTR and DISK algorithms are unable to match feature points correctly in both sets of experiments, which shows that both algorithms are unable to cope with the task of feature point matching for large-scale difference images. In group (g), the LightGlue algorithm obtained good feature point matching results in the second set of experiments, but fewer effective feature point matching pairs were obtained in the first set of experiments, which is due to the fact that LightGlue’s sparse matching local feature point algorithm is unable to accurately measure the ability of its predicted confidence, and thus the matching results are unstable. In the images of group (h), the algorithm proposed in this paper matched enough pairs of feature points and there were no matching errors. More and more accurate feature point matching pairs can be computed to get more accurate image localization and homography matrix, which helps to get visually better ultra-high resolution videos.

#### 4.2.5. Video Synthesis Experiment

**Figure 12:**
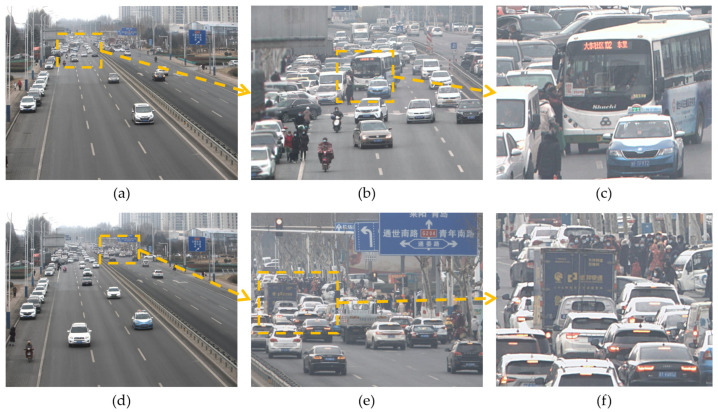
Video synthesis effect. (**a**) Resolution: 20,785 × 15,588; (**b**) Zoom in 5 times; (**c**) Zoom in 8 times; (**d**) Resolution: 20,767 × 15,575; (**e**) Zoom in 5 times; (**f**) Zoom in 8 times.

Video screenshots are shown in Figure 12. Figure 12a,d display the complete video synthesized by the array camera, covering the full view of eight lanes in both directions with a 114° wide-angle video, and the overall visual effect of the video is good. Figure 12b,e show the effect of enlarging by five times the area of high information density in the ultra-high-resolution video. By observing the corresponding areas marked in Figure 12a,d, it can be seen that this area accounts for a smaller proportion in the original ultra-high-resolution video, but contains a large amount of information. Through Figure 12b,e, it can be seen that the enlarged key areas have good clarity. The non-structured embedding algorithm preserves the most valuable field of view areas through sparse coverage. Figure 12c,f continue to zoom in on the key objects in the high information value area, where the text information on the exterior of the bus and the box truck can be clearly seen in the figure. In summary, the algorithm proposed in this paper can synthesize ultra-high-resolution videos in a wide field of view. The synthesized videos have good visual effects, and they have good clarity performance in local key areas.

This section compares several existing array camera architectures from the perspectives of practicality and usability, and the comparison results are recorded in Table 5. The comparison shows that compared with the ultra-high-resolution image synthesis algorithm of single-camera scanning like GigaPan [26], the advantage of the array camera proposed in this paper is that it can synthesize video in real time. Compared with structured array cameras like AWARE-2 [25] and RUSH [43], the advantages of this paper are that there is no need for overlapping fields of view between cameras, the relative positions of the cameras can move, the hardware cost is low, and the algorithm complexity is low. Compared with unstructured array cameras like MGV [32], the advantage of this paper is that the focal length of the camera can change freely. In summary, the method proposed in this paper breaks through the bottlenecks of existing array cameras with a single scale and difficulty to expand, by using a multi-scale scene content correlation mechanism, and realizes the high robustness, easy calibration, and scalability characteristics of unstructured array camera systems.

## 5. Conclusions

To address the issue that a single-lens camera sensor has limited pixels and cannot obtain high-quality detailed textures in large scenes, this paper proposes an unstructured array camera to achieve the synthesis of ultra-high-resolution, ultra-large scene videos. To address the issue of large-scale differences and rotation in array camera image matching and positioning, this paper proposes a feature point extraction algorithm based on symmetric auto-encoding and scale feature fusion, which effectively enhances the feature point extraction performance of array camera images. We propose an image positioning method based on area ratio and homography matrix scaling, which can quickly and accurately calculate the scale ratio of array camera images and position images. At the same time, we propose an image stitching algorithm based on secondary feature matching, which effectively solves the distortion problem of array camera images from different perspectives. In the feature point detection, homography estimation, and feature point matching experiments, good results were achieved compared to several common algorithms. The experimental results show that the algorithm proposed in this paper can synthesize billion-pixel videos in real time, demonstrating its practicality and robustness. In summary, the advancement brought forth by our algorithms is quantitatively significant. The performance of feature point detection has been enhanced by an average of 4.9%, and homography estimation accuracy has improved by an average of 2.5%. Moreover, our approach has successfully reduced the computational load by 18% and decreased the number of network model parameters by 47%, underpinning the efficiency of our method. Notably, the synthesis of billion-pixel videos has been achieved, showcasing the practicality and robustness of our algorithms in handling large-scale data. These improvements underscore the potential of our approach for real-world applications where computational efficiency and high performance are paramount.

## 6. Limitations and Future Work

While the proposed algorithms have shown promising results in the synthesis of billion-pixel videos and improved performance metrics, we acknowledge several limitations that warrant future investigation:

1. Robustness in Diverse Conditions: The feature point extraction algorithm may encounter difficulties in scenes with extremely sparse feature points or significant variations in lighting. Further research could explore adaptive thresholding techniques or deep learning models capable of invariant feature detection under such challenging conditions.
2. Hardware Efficiency: Despite the reduction in computational load and model parameters, the current algorithms could be further optimized for deployment on hardware with limited processing capabilities, such as mobile devices or embedded systems. This could involve the development of more efficient network architectures or the application of model quantization and pruning techniques.
3. Real-Time Processing: For certain applications, such as autonomous navigation, real-time processing is crucial. Future versions of the algorithm could leverage edge computing paradigms or dedicated hardware accelerators like FPGAs or GPUs to meet these stringent latency requirements.

The versatility of our algorithms opens up numerous applications across various fields. Some potential applications include:

1. Digital Heritage Preservation: Our algorithms can be employed to create detailed digital archives of historical sites and artifacts, where capturing texture details is critical. By synthesizing high-resolution representations of these sites, our method could aid in their conservation and study.
2. Autonomous Vehicle Navigation: The robust feature detection and homography estimation capabilities of our algorithms could be leveraged in the development of advanced driver-assistance systems (ADASs) to produce detailed and accurate maps for safe vehicle navigation.
3. Telemedicine: In medical imaging, especially in areas such as dermatology or pathology, our algorithms could provide high-resolution stitched images from an array of lower-resolution images, aiding in diagnosis and allowing for teleconsultation across distances.

By addressing these areas, we not only recognize the current state of our research, but also chart a course for its evolution, ensuring that our work continues to contribute meaningfully to the field and society.

## Figures and Tables

**Table 1:** Improvements in homography transformation parameters.

Parameter	Original	Improvement
scaling	0.8~1.2	0.3~1.7
rotation	−180°~180°	−15°~15°
horizontal displacement	−0.2~0.2	−0.5~0.5
vertical displacement	−0.2~0.2	−0.5~0.5

**Table 2:** Comparison of parameter quantity and operation quantity.

Methods	Number of Parameters (100 Thousand)	Computation Volume (Billion)
SuperPoint	13.04	6.557
Our	6.91	5.377

**Table 3:** Benchmark feature point detection on HPatches.

Methods	57 Illuminations	59 Viewpoints
FAST	0.576	0.503
Harris	0.620	0.556
Shi	0.606	0.552
SuperPoint	0.653	0.671
Our	0.682	0.707

**Table 4:** Homography estimation accuracy.

Methods	e = 1	e = 3	e = 5
ORB	0.162	0.467	0.564
SIFT	0.498	0.786	0.786
SURF	0.334	0.575	0.719
SuperPoint	0.483	0.836	0.910
Our	0.493	0.880	0.927

**Table 5:** Comparison of different array cameras.

System	Video	Overlapping	Position	Focal Length	Realtime	Complexity	Cost
GiganPan	×	√	Fixed	Fixed	×	Low	Low
AWARE-2	√	√	Fixed	Fixed	×	High	High
RUSH	√	√	Fixed	Fixed	√	High	High
MGV	√	×	movable	Fixed	√	Low	Low
Our	√	×	movable	Variable	√	Low	Low

## Data Availability

Data are contained within the article.
